# A SWI/SNF Chromatin Remodelling Protein Controls Cytokinin Production through the Regulation of Chromatin Architecture

**DOI:** 10.1371/journal.pone.0138276

**Published:** 2015-10-12

**Authors:** Teddy Jégu, Séverine Domenichini, Thomas Blein, Federico Ariel, Aurélie Christ, Soon-Kap Kim, Martin Crespi, Stéphanie Boutet-Mercey, Grégory Mouille, Mickaël Bourge, Heribert Hirt, Catherine Bergounioux, Cécile Raynaud, Moussa Benhamed

**Affiliations:** 1 Institut de Biologie des Plantes, UMR8618 CNRS-Université Paris-Sud XI, Saclay Plant Sciences, Orsay, France; 2 Institut des Sciences du Végétal, UPR2355 CNRS, Saclay Plant Sciences, Gif-sur-Yvette, France; 3 Division of Biological and Environmental Sciences and Engineering, King Abdullah University of Science and Technology KAUST, Thuwal, Saudi Arabia; 4 Institut Jean-Pierre Bourgin, UMR1318 INRA/AgroParisTech, Versailles, France; 5 Pôle de Biologie Cellulaire, Imagif, Centre de Recherche de Gif, CNRS, IFR87, Gif-sur-Yvette, France; Ecole Normale Superieure, FRANCE

## Abstract

Chromatin architecture determines transcriptional accessibility to DNA and consequently gene expression levels in response to developmental and environmental stimuli. Recently, chromatin remodelers such as SWI/SNF complexes have been recognized as key regulators of chromatin architecture. To gain insight into the function of these complexes during root development, we have analyzed Arabidopsis knock-down lines for one sub-unit of SWI/SNF complexes: BAF60. Here, we show that BAF60 is a positive regulator of root development and cell cycle progression in the root meristem via its ability to down-regulate cytokinin production. By opposing both the deposition of active histone marks and the formation of a chromatin regulatory loop, BAF60 negatively regulates two crucial target genes for cytokinin biosynthesis (*IPT3* and *IPT7*) and one cell cycle inhibitor (*KRP7)*. Our results demonstrate that SWI/SNF complexes containing BAF60 are key factors governing the equilibrium between formation and dissociation of a chromatin loop controlling phytohormone production and cell cycle progression.

## Introduction

Plant development relies on the complex interaction of several phytohormones that govern cell division and growth to shape the whole plant body. This intricate network involves tight control of signaling processes including the biosynthesis and degradation of phytohormones, their transport, perception and cellular responses. Cytokinins (CK) are amongst the first phytohormones identified, based on their ability to stimulate cell proliferation *in vitro*. They participate in virtually all aspects of plant development and physiology including the shoot and root meristems, leaf senescence and responses to environmental cues [1,2]. In *Arabidopsis*, adenosine phosphate-isopentenyltransferases (IPTs) are the enzymes responsible for cytokinins production [3]. tRNA IPTs are required for the production of *cis*-zeatin (*c*Z)-type CKs and ATP/ADP-dependent IPTs are required for the production of *N* ^6^-(Δ^2^-isopentenyl) adenine and *trans*-zeatin (*t*Z)-type CKs [3]. It was proposed that the *t*Z-type CKs are the most important forms and are more physiologically active than *c*Z-type CKs in *Arabidopsis* [4].

From a physiological point of view, CKs are generally considered to promote mitotic cell division in the shoot, but differentiation and transition to the endocycle in the root. Indeed, CK overproduction inhibits primary root elongation and impairs lateral root development [5]. It was suggested that CKs control root development through at least two different ways. One would antagonize auxin transcriptional responses and its ability to stimulate cell division [6,7]. The second one may have a more direct effect on the root cell cycle and is independent of auxin signaling. One of the underlying mechanisms involves ARR2 (a type-B ARR) and *CCS52A1*, a gene involved in the control of endoreduplication [8]. In response to CK, ARR2 directly binds to the promoter of *CCS52A1* to activate its transcription at the junction between the elongation and differentiation zones in order to stimulate the degradation of mitotic cyclins and induce endoreduplication [8]. Despite all the available data regarding the key role of CK signaling in the control of root development and cell cycle progression, little is known about how *IPT* expression, the enzyme responsible for the rate limiting step for CK production, is regulated. Until now, only the HD-ZIPIII transcription factor PHABULOSA (PHB) was described as a regulator of *IPT7* [9].

Chromatin modifications are crucial components of the regulation of gene expression, and there is accumulating evidence that they participate in hormonal signaling. For example, the formation of a chromatin loop has been recently described to modulate the expression dynamics of the auxin-responsive gene PID (Ariel et al, 2014) demonstrating that chromatin modifications are elements of cellular responses to phytohormones. Reciprocally, phytohormone production can be influenced by chromatin changes: the BRAHMA (BRM) and SWI3C sub-units of the SWI/SNF chromatin remodeling complex (CRC) are required for normal expression of genes involved in gibberellin biosynthesis [10,11]. As described in detail previously [12], CRCs are multi-subunit complexes that use the energy of ATP hydrolysis to modify DNA-histone interactions and alter the location or conformation of nucleosomes [13]. These multi-protein complexes control access to chromatin DNA by regulating the structure, the type of histone variants and nucleosome positioning. The distinctive feature of CRCs is the presence of a central ATPase, belonging to the SWI2/SNF2 family [14,15]. As described in detail previously [16] *Arabidopsis* displays four SWI2/SNF2 ATPases (BRM, SPLAYED, CHR12, and CHR23) and two SWI/SNF ASSOCIATED PROTEINS 73 (SWP73A/CHC2 and SWP73B/CHC1 also called BAF60) (The Chromatin Database, www.chromdb.org; [12,17,18,19,20]. In addition to their primary activity that enables them to alter histone/DNA interactions to allow access of sequence-specific binding proteins to the genomic DNA [21], CRCs also impinge on various other chromatin-related processes. Indeed, histone modifications such as acetylation, methylation, phosphorylation and ubiquitination are usually linked to chromatin remodeling and are also extremely important for regulation of gene expression [22]. Furthermore, it was proposed that this kind of complexes can also modify the formation of chromatin loops to modulate gene expression [12,23,24].

In this work, we demonstrate that BAF60 is a positive regulator of root growth and root meristem size through its capacity to control CK production and cell cycle progression. We show that BAF60 directly inhibits the deposition of active histone marks and controls the formation of a chromatin regulatory loop on two crucial target genes, the CK biosynthesis genes *IPT3* and *IPT7*. Furthermore, BAF60 also target the CK-regulated cell cycle inhibitor *KRP7* demonstrating a complex regulation of CK response during root development.

## Materials and Methods

### Plant Material and Growth Conditions

*Arabidopsis* seeds of RNAi lines BAF60 (CS23961), in the Wassilewskija (Ws) background, were obtained from the Nottingham Arabidopsis Stock Centre (NASC). Seeds were surface-sterilized by treatment with bayrochlore for 20 min, washed, and imbibed in sterile-water for 2–4 days at 4°C to obtain homogeneous germination. Plants were grown vertically in chambers at 20°C on sterile half-strength Murashige and Skoog (MS) medium and agar (0.8% w/v) in long-day (16h of light at 20°C, 8h of darkness at 18°C) conditions. For root growth assays, plants were grown vertically. Plates were scanned at 3 or 4 days intervals, and root length was measured using the Image J software (http://rsb.info.nih.gov/ij/).

For ChIP and qPCR assays, plants were grown on nylon membranes, and roots were cut with razor blade, crosslinked or not, frozen in liquid nitrogen and stored at -80°C.

### Confocal Imaging

The cell wall of the 14-day-old roots was stained according to Truernit et al, 2008. Root tips were recorded with a SP2 confocal microscope (Leica Microsystem GmbH). Serial optical sections were reconstituted into 3D image stacks and measurements where done in imageJ (http://rsb.info.nih.gov/ij/). Meristematic zone length was defined along a cortex cell file as the distance from the QC until the cortex cells started to elongate (length of the cell at least twice the size of the previous one). Measures per root are the average of two opposite cell files.

### Mitotic Index

7-day-old seedlings were in paraformaldehyde (4% w/v) for fixed 20 min, washed in 1x PBS pH 7.4 and stained with Hoescht (supplied in Click-iT kit and diluted 2000 fold in 1x PBS pH 7.4, Life Technologies) during 30 min. Roots tips were mounted on slides in water under cover slips and squashed with a cone on a glass plate. Mitotic events per root tip were counted using an epifluorescence microscope (SVII; Zeiss).

### Histochemical Staining of GUS Activity

After 15-min fixation in 100% cold acetone, β-glucuronidase (GUS) activity was revealed as described previously [25]. After 1 h at 37°C, samples were washed in 70% ethanol, fixed with PFA during 20 min under vacuum, and then cleared using a chloral hydrate solution overnight at room temperature (8 g of chloral hydrate (Sigma), 2 ml of 50% glycerol and 1 ml of water). Images were captured on a macroscope (AZ100, NIKON) with a video camera Nikon RI1.

### EdU Incorporation Assay

7-day-old seedlings were incubated in 0.5x MS medium liquid medium (Basal Salt Mixure M0221, Duchefa) supplemented with 10 μm EdU (Life Technologies) for 3 h in a 6-wells plate with shaking, in a long-day (16h light, 8h night, 20°C) growth chamber. Plantlets were fixed with 3.7% paraformaldehyde in 1x PBS pH 7.4 during 15mn at room temperature and washed twice with PBS-BSA (1x PBS pH 7.4, BSA 3% w/v). Plantlets were then permeabilized in PBS Triton (1x PBS pH 7.4, Triton 0.5% w/v) for 20 min at room temperature. Plantlets were washed twice in PBS-BSA prior to the Click-iT reaction. The Click-iT reaction mix for EdU visualization was prepared according to the manufacturer's instructions (Click-iT™ EdU AlexaFluor 647 Imaging Kit, Life Technologies), and the reaction was performed in the dark for 30 min at room temperature. Nuclei were counterstained with Hoechst (supplied in Click-iT kit and diluted 2000 fold in 1x PBS pH 7.4). Roots were mounted on slides in water under cover slips and squashed with a cone. Observations were done with an epifluorescence microscope (SVII; Zeiss), and images were captured with a color charge-coupled device camera (Power HAD; Sony).

### Flow Cytometry

7-day-old root tips were cut with razor blade and protoplasts were obtained by incubating in an enzymatic solution (cellulose 15 mg/mL, pectolyase 1mg/mL, 10mM KCl, 2mM MgCl2, 2mM CaCl2, 1mg/mL BSA, 0.4 mg/mL MES, 0.1 mg/mL sorbitol) for 1h at 22°C. Protoplasts were washed in 10mM KCl, 2mM MgCl2, 2mM CaCl2, 1mg/mL BSA, 0.4 mg/mL MES, 0.1 mg/mL sorbitol and resuspended in Galbraith’s buffer supplemented with 0,1% Triton X-100, 1% polyvinylpyrrolidone 10,000, 5 mM metabisulfite, and 5 mg/mL RNase from a stock solution at 50 units/mg. Extracts were filtered on 48μm pore nylon, and released nuclei were stained with propidium iodide 50 μg/mL. DNA content of nuclei was determined using a Cyflow SL flow cytometer (Partec) with a 532nm solid state laser (30mW) excitation and emission collected after a 630/30 nm filter.

### Zeatin Quantification

100 mg of fourteen-day-old roots were cut with razor blade and immediately frozen in liquid nitrogen. Roots were ground and lyophilized 48h to obtain 10 mg of final dry matter. For each sample, 10 mg of freeze-dried powder were extracted with 0.8 mL of acetone/water/acetic acid (80/19/1 v:v:v). For the cytokinins, stable labelled isotopes (OLChemIm) were used as internal standards and added as follows: 1 ng of ^2^H_5_-t-Z7G (*trans*-zeatin-7-glucoside), 1 ng of ^2^H_5_-t-Z9G (*trans*-zeatin-9-glucoside), 1 ng of ^2^H_5_-t-ZOG (*trans*-zeatin O-glucoside), 1 ng of ^15^N-t-Z (*trans*-zeatin), 1 ng of ^2^H_5_-t-ZROG (*trans*-zeatin riboside O-glucoside), 1 ng of ^2^H_5_-t-ZR (*trans*-zeatin riboside), 1 ng of ^2^H_5_-t-ZRMP (*trans*-zeatin riboside monophosphate),. The extract was vigorously shaken for 1min, sonicated for 1 min at 25 Hz, shaken for 10 minutes at 4°C in a Thermomixer (Eppendorf^R^), and then centrifuged (8,000xg, 4°C, 10 min). The supernatants were collected, and the pellets were re-extracted twice with 0.4 mL of the same extraction solution, then vigorously shaken (1 min) and sonicated (1 min; 25 Hz). After the centrifugations, the three supernatants were pooled and dried (Final Volume 1.6 mL).

Each dry extract was dissolved in 140 μL of acetonitrile/water (50/50; v/v), filtered, and analyzed using a Waters Acquity ultra performance liquid chromatograph coupled to a Waters Xevo Triple quadrupole mass spectrometer TQS (UPLC-ESI-MS/MS). The compounds were separated on a reverse-phase column (Uptisphere C18 UP3HDO, 100*2.1 mm*3μm particle size; Interchim, France) using a flow rate of 0.4 mL min^-1^ and a binary gradient: (A) acetic acid 0.1% in water (v/v) and (B) acetonitrile with 0.1% acetic acid. For cytokinins the solvent gradient was applied as follow (t., % A): (0 min., 95%), (12 min., 40%), (13 min., 0%), (16 min., 95%) and the column temperature was 40°C. Mass spectrometry was conducted in electrospray and Multiple Reaction Monitoring scanning mode (MRM mode), in negative ion mode. Relevant instrumental parameters were set as follows: capillary 1.5 kV (negative mode), source block and desolvation gas temperatures 130°C and 500°C, respectively. Nitrogen was used to assist the cone and desolvation (150 L h^-1^ and 800 L h^-1^, respectively), argon was used as the collision gas at a flow of 0.18 mL/min. Samples were reconstituted in 140 μL of 50/50 acetonitrile/H_2_O (v/v) *per* mL of injected volume. The limit of detection (LOD) and limit of quantification (LOQ) were extrapolated for hormone from calibration curves and samples using Quantify module of MassLynx software, version 4.1.

### RNA Extraction and Real Time Quantitative PCR Analysis

Total RNA were extracted from roots with the RNeasy MiniPrep kit (Macherey), according to the manufacturer's instructions. First strand cDNA was synthesized from 2μg of total RNA using Improm-II reverse transcriptase (A3802, Promega) according to the manufacturer’s instructions. 1/25th of the synthesized cDNA was mixed with 500nM of each primer and LightCycler® 480 Sybr Green I master mix (Roche Applied Science) for quantitative PCR analysis. Products were amplified and fluorescent signals acquired with a LightCycler® 480 detection system. The specificity of amplification products was determined by melting curves. *UBQ10* was used as internal control for signals normalization. Exor4 relative quantification software (Roche Applied Science) automatically calculates relative expression level of the selected genes with algorithms based on ΔΔCt method. Data were from duplicates of at least three biological replicates. All the sequences of primers used are given in S1 Table.

### Chromatin Immuno-Precipitation Analysis

ChIP assays were performed on 14-day-old in vitro roots using anti-GFP (Clontech Cat # 632592), anti-IgG (Millipore), anti-H3K27me3 (Millipore), anti-H3K4me3 (Abcam) and RNA Polymerase II (Abcam) antibodies, modified from [26]. Briefly, after plant material fixation in 1% (v/v) formaldehyde, tissues were homogenized, nuclei isolated and lysed. Cross-linked chromatin was sonicated using a water bath Bioruptor UCD-200 (Diagenode, Liège, Belgium) (15s on/15s off pulses; 15 times). The complexes were immunoprecipitated with antibodies, overnight at 4°C with gentle shaking, and incubated for 1 h at 4°C with 50 μL of Protein AG UltraLink Resin (Thermo scientific). Immunoprecipitated DNA was then recovered using the IPure kit (Diagenode, Liège, Belgium) and analysed by quantitative real-time PCR. An aliquot of untreated sonicated chromatin was processed in parallel and used as the total input DNA control.

### Chromosome Conformation Capture

Two grams of 14-day-old roots were cross-linked in 1% (v/v) formaldehyde at room temperature for 20min. Cross-linked plantlets were ground and nuclei were isolated and treated with SDS 0,3% at 65°C for 40min. SDS was sequestered with 2% Triton X-100. Digestions were performed overnight at 37°C with 400U NheI-HF (NEB) for the *IPT3* locus, 400U BglII (NEB) for the *IPT7* locus or 400U MfeI (NEB) for the *KRP7* locus. Restriction enzymes were inactivated by addition of 1,6% SDS and incubation at 65°C for 20min. SDS was sequestered with 1% Triton X-100. DNA was ligated by incubation at 22°C for 5h in 5mL volume using 100U of T4 DNA ligase (Fermentas). Reverse crosslinking was performed by overnight treatment at 65°C. DNA was recovered after Proteinase K treatment by phenol/chloroform extraction and ethanol precipitation. Relative interaction frequencies were calculated by quantitative real-time PCR using 15 ng of DNA. A region uncut by NheI-HF, BglII or MfeI was used to normalize the amount of DNA.

### Accession Numbers and Lines

*BAF60* (AT5G14170), *IPT3* (AT3G63110), *IPT7* (AT3G23630), *KRP7* (AT1G49620).

## Results

### BAF60 Controls Root Development

**Fig 1:**
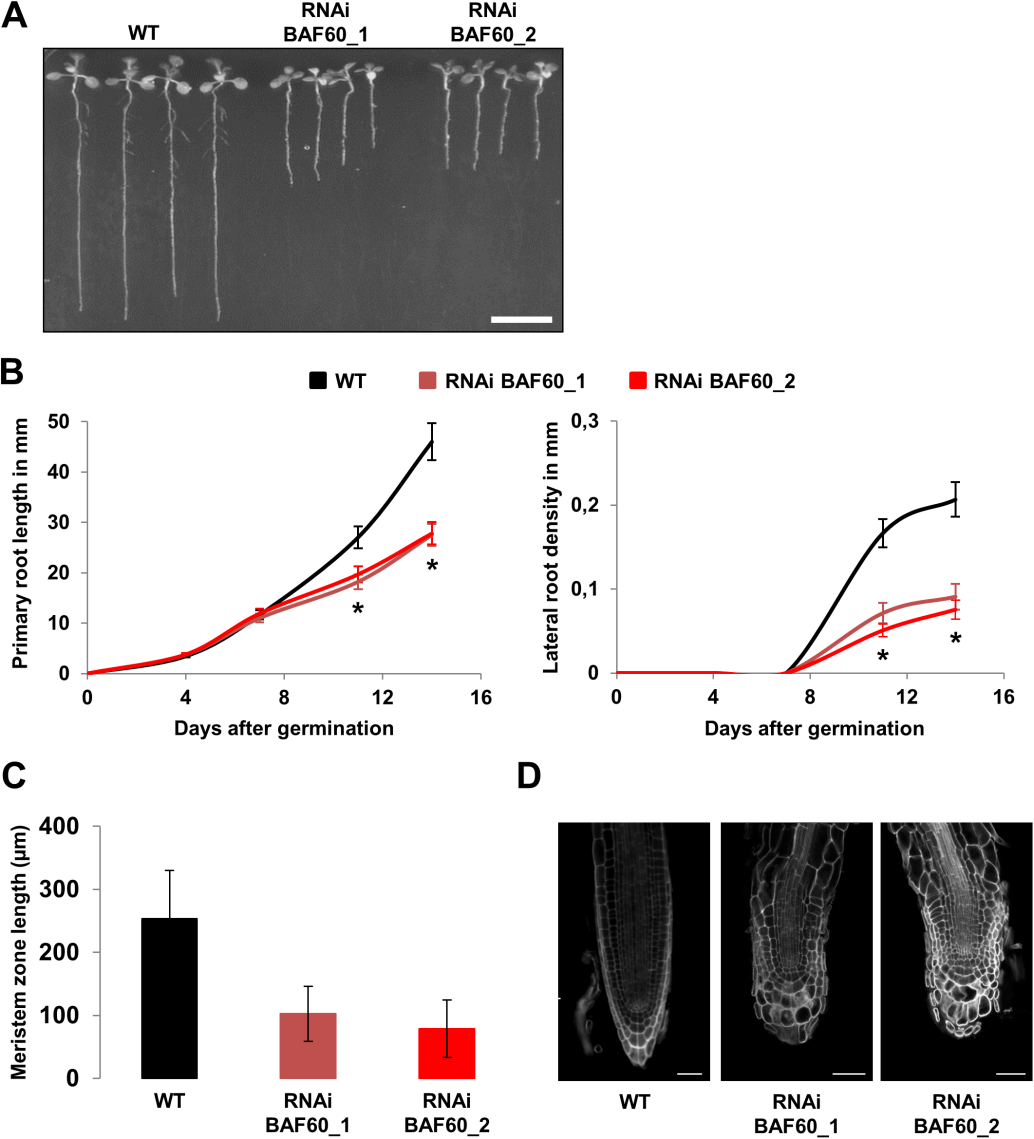
BAF60 regulates root development. (A) Fourteen-day-old wild type (WT) and *BAF60* RNAi lines grown vertically under LD conditions. Bar = 10 mm. (B) Time course analysis of root length (left) and lateral root density (right) in wild type (black) and *BAF60* RNAi plantlets (red) grown vertically on MS medium. Values are average +/- standard deviation (n = 100). Asterisks indicate significant difference from the wild type (WT) (P < 0.01, Student’s t test). (C) Quantification of root meristem length of fourteen-day-old wild type and *BAF60* RNAi lines grown vertically on half MS medium. Values are average +/- standard deviation (n > 15). (D) Confocal images of mPS-PI–stained root tips of fourteen-day-old wild type and *BAF60* RNAi plantlets growth vertically on MS medium. Bars = 50 μm.

To characterize the role of BAF60 during root development we analyzed the root phenotype of the previously described RNAi lines BAF60 [12,27]. Interestingly, they displayed a short root phenotype and a reduction of lateral root number (Fig 1A and 1B). A time-course analysis revealed that growth of the BAF60 RNAi roots resembled that of the wild-type during the first 7 days after germination (DAG) (Fig 1B), upon which the elongation of the BAF60 RNAi primary roots became gradually slower compared to the wild-type (Fig 1B). To determine more precisely the cause of this reduction in root growth, we measured the size of the root meristematic zone 5 and 14 DAG, time-points corresponding to before and after the moment when the growth reduction became obvious. We observed that this specific zone was unchanged at early stages of development (S1 Fig), but became significantly shorter in the RNAi line compared to the WT at 14 DAG (Fig 1C), indicating a gradual loss of the division activity in this zone. In addition, the root tip of *BAF60* RNAi lines appeared affected with enlarged and poorly aligned cells (Fig 1D).

**Fig 2:**
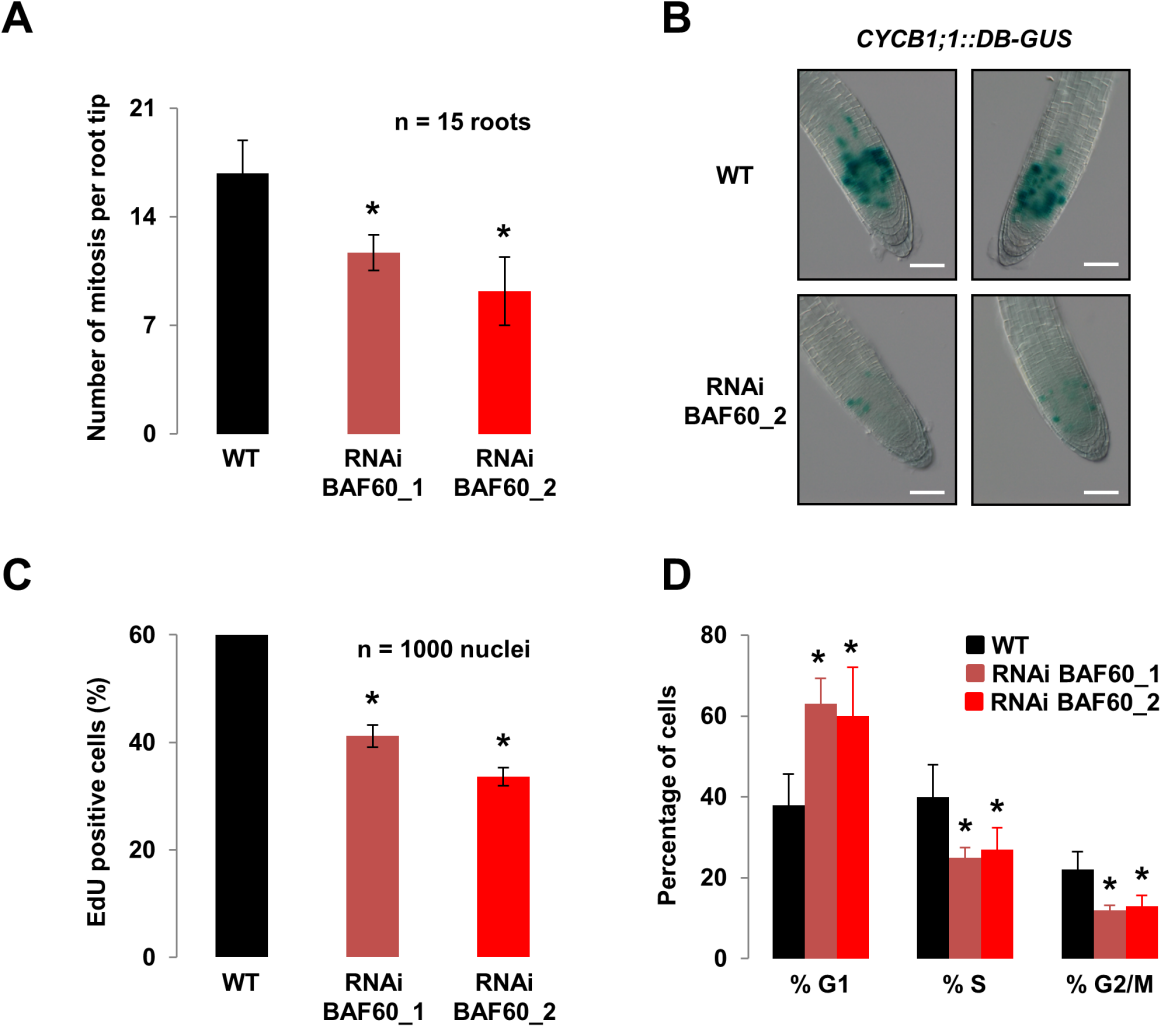
BAF60 control cell cycle progression. (A) Mitotic index in root tips of 7-day-old wild-type and *BAF60* RNAi plantlets grown vertically on MS medium. Values are average +/- standard deviation of biological triplicates. Asterisks indicate significant difference from the wild type (WT) (P < 0.01, Student’s t test). (B) Histochemical staining of the GUS activity in wild-type and *BAF60_2* RNAi roots harbouring the CYCB1;1::DB-GUS construct. The number of stained cells reflects the number of cells in G2/M. Plantlets were grown vertically for 7 days on MS medium. Bars = 100 μm. (C) Proportion of EdU positive cells in the root tips of seven-day-old wild-type and *BAF60* RNAi lines grown vertically on MS medium. Values are average +/- standard deviation of biological triplicates. Asterisks indicate significant difference from the wild type (WT) (P < 0.01, Student’s t test). (D) Cell cycle progression was examined by flow cytometry. Histograms show the percentage of cells in G1, S and G2/M phases in wild-type and *BAF60* RNAi root-derived protoplasts. Plantlets were grown vertically for 7 days on MS medium before protoplast preparation. Data presented are representative of three biological replicates. Asterisks indicate significant difference from the wild type (WT) (P < 0.05, Student’s t test).

To assess if this phenotype could result from a mis-regulation of cell cycle progression, we analyzed cell cycle progression 7 DAG, the time corresponding to the onset of growth retardation in BAF60 RNAi lines. We found a reduction in the number of mitosis (Fig 2A), in the number of G2/M cells measured using the *CYCB1;1*:*GUS* reporter line [28] (Fig 2B), as well as in the DNA replication activity measured via EdU incorporation (Fig 2C). These results were corroborated by RT-qPCR analysis, which showed a clear reduction in *CYCB1;1* levels in *BAF60* RNAi lines (S2 Fig). Altogether, these observations suggested that the cell cycle was blocked at the G1 phase. To confirm this, we analyzed the percentage of cells in the different phases in the root tip of RNAi and WT lines by flow cytometry. We confirmed that the knock-down of BAF60 resulted in an increased proportion of G1 cells (Fig 2D).

### BAF60 Impacts Cytokinin Production

**Fig 3:**
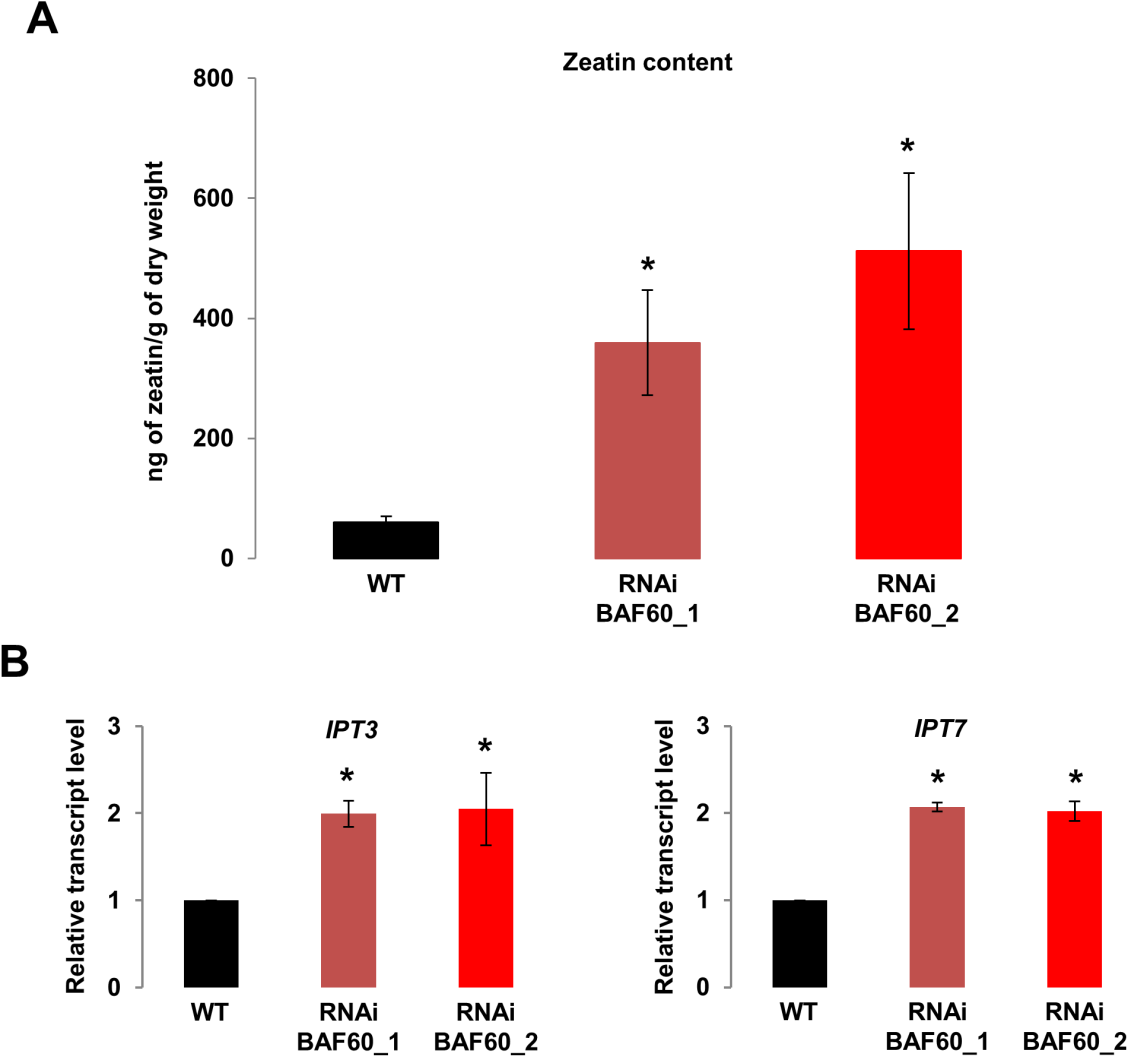
BAF60 regulates cytokinin production. (A) Zeatin content in the roots of 14-day-old wild-type and *BAF60* RNAi plantlets grown vertically on MS medium. Values are average +/- standard deviation of four biological replicates. Asterisks indicate significant difference from the wild type (WT) (P < 0.01, Student’s t test). (B) qRT-PCR data show the relative expression of the indicated genes in wild-type and *BAF60* RNAi lines. Total RNA samples were collected from 14-day-old roots. mRNA abundance was quantified by qRT-PCR and expressed relative to the abundance of *UBQ10* transcripts. Values are average +/- standard deviation of triplicates. Data presented are representative of three biological replicates. Asterisks indicate significant difference from the wild type (WT) (P < 0.01, Student’s t test).

CK are a family of phytohormones well known for their important role as regulators of plant root development (reviewed in [29] [2]). It was shown that CK inhibit root growth and branching [30,31], and more specifically that a reduction of cytokinin accumulation increases meristematic cell number in the root [32]. We therefore hypothesized that the short root phenotype observed in RNAi lines could be due to an over production of CK. To test this hypothesis, we measured the CK levels in roots of both WT and *BAF60* RNAi lines at 14 DAG, and observed a 5 to 7 fold increase in zeatin in the *BAF60* RNAi lines (Fig 3A). This increase in zeatin accumulation was accompanied by a decrease in the amount of inactive N-glucosylated forms of CK (zeatin 7 glucoside and zeatin 9 glucoside) (S3 Fig). These results suggested that BAF60 is involved in the regulation of CK biosynthesis and/or metabolism. Given its role as a sub-unit of chromatin remodeling complexes, one hypothesis would be that BAF60 could target genes involved in CK production. The rate limiting step of zeatin biosynthesis is catalyzed by the ATP/ADP isopentenyltransferase (IPT) enzyme, and nine isoforms of this enzyme exist in Arabidopsis (IPT1-9) [3]. Among 7 *ITP* genes tested, *IPT3* and *IPT7* were induced in *BAF60* RNAi lines (Fig 3B and S4 Fig), suggesting that overexpression of these genes leads to the higher quantity of zeatin observed in BAF60 RNAi lines.

### BAF60 Controls the Expression of *IPT3* and *IPT7* by Regulating Histone Modifications

**Fig 4:**
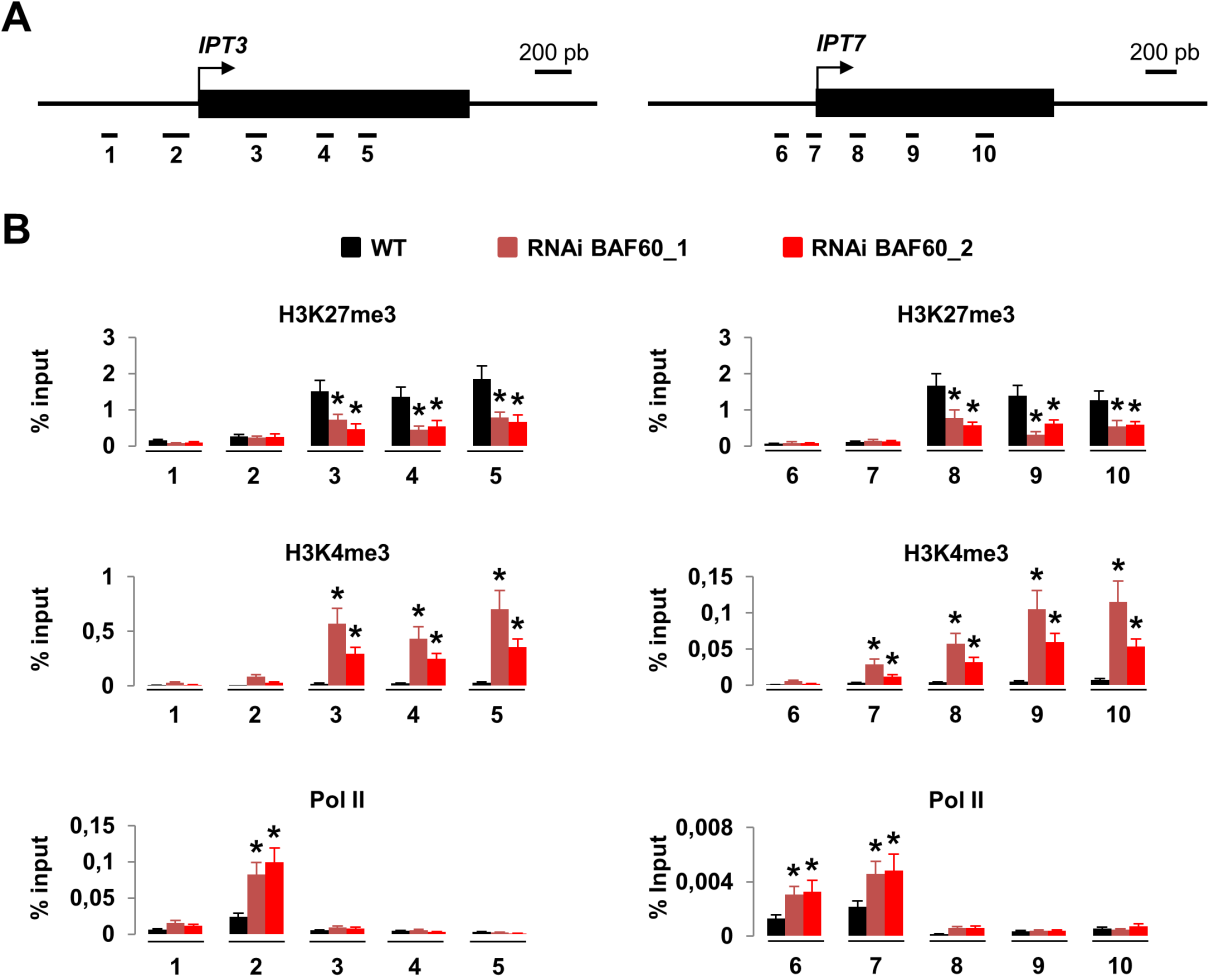
BAF60 controls histone modifications and RNA Pol II occupancy at the *IPT3* and *IPT7* loci. (A) Schematic representation of the regions of the *IPT3* and *IPT7* loci analysed. Black boxes correspond to exons, the arrow indicates the site of translation initiation, numbers indicate the positions of primer pairs used. (B) Quantification data of the chromatin immunoprecipitation results. Nuclei were extracted from 14-day-old roots and immunoprecipited with antibodies specific for H3K27me3, or H3K4me3 or RNA polymerase II. Average relative quantities ± sd are shown for each sample. Data [12]significant difference from the wild type (WT) (P < 0.05, Student’s t test).

To dissect the molecular mechanisms underlying the over-expression of *IPT3* and *IPT7* in *BAF60* RNAi plants, we analyzed by chromatin immunoprecipation (ChIP) the histone modifications induced by the knock down of BAF60. Both a transcriptional repressive mark (H3K27me3) and a transcriptional activating mark (H3K4me3) were assessed using primers covering 5 specific regions of each of the *IPT3* and *IPT7* loci (Fig 4A). In *BAF60* RNAi plants, H3K27 tri-methylation was decreased while H3K4me3 was increased on these loci, compared to the WT (Fig 4B). To assess the effect of the increase in H3K4me3 enrichment on RNA Pol II occupancy, ChIP assays were performed using an antibody raised against RNA Pol II. We observed that RNA Pol II was present on the proximal promoter of *IPT3* and *IPT7* in both genotypes but with a 2-fold increase in *BAF60* RNAi plants (Fig 4B). These results are consistent with the observed *IPT3 and IPT7* up-regulation caused by down-regulation of BAF60. Altogether our results suggest that BAF60 inhibits H3K4me3 deposition and RNA pol II loading and promotes H3K27me3 deposition at the *IPT3 and IPT7* loci to regulate their expression level.

### BAF60 Binds the *IPT3* and *IPT7* Loci and Regulates Loop Formation at These DNA Regions

**Fig 5:**
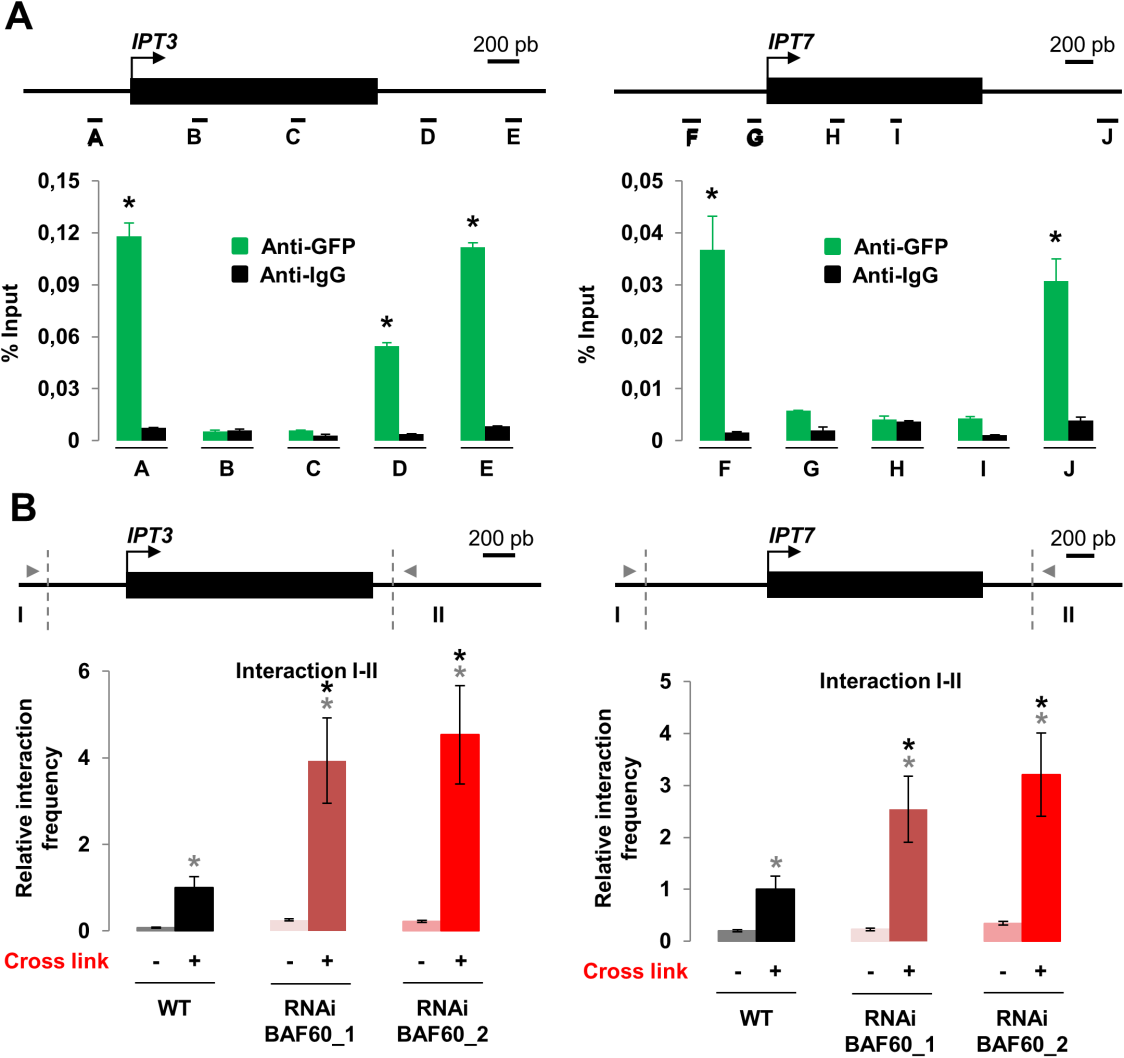
BAF60 binds *IPT3* and *IPT7* loci to regulate gene loops formation. (A) Top: Schematic representation of the *IPT3* and *IPT7* loci, the position of each primer pair used for ChIP-qPCR is indicated. The arrow indicates the position of the transcription start site. Exons are represented as black boxes and introns as black lines. Bottom: Quantification data of chromatin immunoprecipitation results. Nuclei were extracted after cross-linking from 14-day-old roots expressing the BAF60-CFP transgene. Chromatin-protein complexes were isolated and immuno-precipitated with antibodies specific for GFP or IgG. Average relative quantities ± sd are shown for each sample. Data presented are representative of three biological replicates. Asterisks indicate significant difference from the wild type (WT) (P < 0.05, Student’s t test). (B) Quantitative 3C of the *IPT3* and *IPT7* loci using region I as the anchor region in 14-day-old WT and *BAF60* RNAi roots. Relative interaction frequencies were calculated as described in Materials and Methods. Data are the average of three biological replicates (each performed on three technical replicates). In the graph, NheI-HF and BglII restriction sites are indicated with vertical dotted lines for *IPT3* and *IPT7* respectively. A schematic representation of these loci is shown above with the position of primers used for the 3C analysis represented by grey arrowheads. Grey Asterisks indicate significant difference between the crosslinked and the not crosslinked (P < 0.05, Student’s t test). Black Asterisks indicate significant difference from the wild type (WT) (P < 0.05, Student’s t test).

To determine if *IPT3* and *IPT7* are direct targets of BAF60, we examined the ability of a BAF60-CFP fusion to bind to the *IPT3 and IPT7* loci by ChIP using an anti-GFP antibody [12]. A ChIP assay using antibodies against Immunoglobulin G (IgG) was used as a negative control. A specific association of BAF60 with the promoter regions of *IPT3 and IPT7* as well as the 3’ region of these loci was observed (Fig 5A). These results showed that BAF60 binds to *IPT3 and IPT7* to directly control their expression.

We next asked what could be the effect of BAF60 binding on these loci. Recently, we demonstrated that BAF60 controls the formation of a gene loop at the *FLC* locus [12]. To determine if BAF60 could act similarly on the *IPT3* and *IPT7* loci, we performed chromosome conformation capture (3C) experiments. As a control, we verified that no PCR product was observed when our plant material was not cross-linked (Fig 5B). Interestingly, we observed a gene loop structure on both loci in the WT, and these loops were at least twice more present in the *BAF60* RNAi context. These results suggest that BAF60 plays a negative role in loop formation and/or stabilization at these loci. Altogether our results suggested that BAF60 controls the production of CK by repressing at the transcriptional level the expression of *IPT3* and *IPT7* genes through the repression of the formation of a gene loop.

### BAF60 Directly Regulates the Epigenetic Landscape of the Cell Cycle Inhibitor *KRP7*

CK treatment has been reported to promote early differentiation of root meristem cells resulting in a premature increase in DNA content [8], which is in sharp contrast with the observed G1 block in the root tip of *BAF60* RNAi lines. In mammalian cells, BAF60 directly controls the expression of cell cycle genes [33]. Moreover, CK are known to induce the expression of the cyclin dependent kinase inhibitors Kip-related Protein 7 (KRP7,[34]). Quantification of the expression level of this specific KRP in *BAF60* RNAi lines revealed that KRP7 was over-expressed compared to the WT (S5 Fig). To determine whether this up-regulation was an indirect consequence of CK over-accumulation or whether it could result from direct binding of BAF60 to the KRP7 locus, we performed ChIP-qPCR analysis in the BAF60–CFP lines. Interestingly, BAF60 bound to the 5’ and 3’ regions of KRP7 (S6 Fig) similarly to the *IPT3* and *IPT7* loci. Furthermore, although H3K27 tri-methylation was not detectable in either background (data not shown), H3K4me3 levels were increased in *KRP7* locus in *BAF60* RNAi lines (S7 Fig). Consistently, RNA Pol II occupancy was increased in *BAF60* RNAi line (S7 Fig). Altogether our results suggest that BAF60 inhibits H3K4me3 deposition and RNA pol II recruitment at the *KRP7* locus to promote its repression.

Finally, to determine if BAF60 could also control the formation of a gene loop structure at the KRP7 locus, we performed 3C experiments. Interestingly, we observed that the *KRP7* loop was about 3 times more present in the *BAF60* RNAi context (S8 Fig), suggesting that BAF60 plays a negative role in loop formation and/or stabilization at the *KRP7* locus.

Altogether our results suggest that BAF60 controls cell cycle progression by modulating chromatin architecture and epigenetic patterns of the *IPT3* and *IPT7* CK production genes and the KRP7 cell cycle inhibitor at transcriptional level.

## Discussion

Despite intensive biochemical and genetic studies of ATP-dependent chromatin remodelling complexes in yeast, drosophila and mammalian cells, the function of these complexes in developmental processes of higher eukaryotes remains largely unknown. To dissect the role of SWI/SNF remodelling complexes in plants, we analyzed the molecular function of one accessory subunit, BAF60, a homolog of the yeast SWP73 protein. In this study, we showed that BAF60 is a positive regulator of root growth and root meristem size. We also found that BAF60 controls CK production and cell cycle progression, via a direct role on histone mark deposition and chromatin architecture spanning two crucial CK genes, *IPT3* and *IPT7*, and the cell cycle negative regulator *KRP7*.

CK are very important hormonal regulators of root development, and they have long been known to affect lateral root formation [35,36,37]. CK are also negative regulators of primary root growth [38,39], and affect the size of the root apical meristem [32], mainly by controlling the transition from cell proliferation to differentiation [8]. Indeed, the CK responsive factor ARR2 regulates the expression of the *CCS52-1* gene, which encodes an activator of the Anaphase Promoting Complex/Cyclosome involved in the degradation of mitotic cyclins [40]. In addition, CK promote cell proliferation in the quiescent center via their action on auxin distribution in the root tip [41].

Even though the molecular mechanisms underlying the function of CK during root development are beginning to be well described, the transcriptional mechanisms that control CK production remain very poorly understood. The HD-ZIPIII transcription factor PHABULOSA (PHB) is the only known transcriptional regulator of genes involved in CK biogenesis. In fact PHB was shown to directly activate the CK biosynthesis gene *ISOPENTENYL TRANSFERASE 7* (*IPT7*) [9], thus promoting cell differentiation and regulating root length. Surprisingly, the authors also found that CK in turn negatively regulate the expression of both PHB and its negative regulator mir165 and suggested that this complex feedback mechanism may be required for robust CK dependent regulation of root development. In this work, we identified BAF60 as a new component regulating the accumulation of CK by directly targeting the chromatin status of two *IPT* genes.

Indeed, *IPT3* and *IPT7* are over-expressed in BAF60-RNAi lines and BAF60 binds directly to these loci. A connection between SWI/SNF complexes and CK signaling was recently uncovered [42] but nothing is known about their action in CK biosynthesis: BRM was shown to interact with TCP4 to activate the expression of genes involved in CK response, including the *ARR16* gene, an A-class ARR factor that inhibits CK signaling. In fact, *brm* mutants were shown to be over-sensitive to CK, consistent with its action on negative regulators of CK signaling. However, they did not test if the over-sensitivity of *brm* to CK could not at least partly be due to changes in CK accumulation or biosynthesis. Because BAF60 and BRM can be part of the same protein complexes [16], it is likely that ATP-dependent chromatin remodeling complexes act on CK signaling both by modulating CK levels and perception.

In the root meristem, the balance between auxin and CK signaling controls the transition from cell proliferation to differentiation, with auxin stimulating division and preventing elongation and CK having an opposing function [8,20], and the two hormones act on cell cycle gene expression. In this study, we show that BAF60 directly binds to the *KRP7* gene encoding a CDK inhibitor to regulate its expression, thus providing evidence for an additional layer of regulatory complexity. The consecutive over-expression of KRP7 in *BAF60* RNAi lines could account for the increase in the number of G1 cells. Indeed, high levels of ICK/KRP proteins have been shown to inhibit the G1-to-S phase transition of both mitotic and endocycling cells [43]. Interestingly, it was shown that CK can induce *KRP7* expression [34], raising the question of the primary cause of *KRP7* mis-regulation in *BAF60* RNAi lines: is it due to the over accumulation of CK or to a direct effect of BAF60 on this locus? From our data and the available literature the answer is probably both. On one hand, exogenous CK is sufficient to induce over-accumulation of the *KRP7* transcript [34]. On the other hand, we showed that BAF60 binds the *KRP7* locus to control its transcriptional status through modulation of its chromatin structure and the deposition of RNA polymerase II and specific histone marks. Altogether these data suggested that both CK and BAF60 regulate the expression of *KRP7*, linking chromatin remodelers to CK signaling and cell cycle regulation. In Arabidopsis, a connection between chromatin organization and auxin has been recently described [44]: it was proposed that auxin modulates a chromatin loop through the action of a long non coding RNA on the *PID* locus to regulate its transcription. Reciprocally, here we show that changes in chromatin structure can in turn regulate hormone production. Although available data concerns only a few genes, it is likely that these findings can be generalized to many loci, and that more global analyses of BAF60 targets will provide evidence for a complex interplay between hormone signaling and chromatin organization. Hormones may modulate chromatin structure, while chromatin structure can also modulate hormone levels *in planta*.

From a molecular point of view, we showed that BAF60 could directly repress *IPT3*, *IPT7* and *KRP7* transcription by modulating chromatin structure at these loci. Genome topology has emerged as a key player in genome functions. In eukaryotes, gene expression is influenced by the interactions between transcription factors and the DNA, which can adopt diverse 3D chromatin structural conformations [45]. The transcription capacity of genes is determined by the dynamics of chromatin structure, which play a key regulatory role in gene expression by directly influencing DNA accessibility [46]. Chromatin organization and structure can lead to both local and long-distance gene loop formation, structurally positioning distant gene sequence elements in close proximity. Local chromatin loops joining 5′ and the 3′ ends of a single gene are proposed to allow an efficient recycling of the RNA polymerase II (RNA Pol II) from the termination site back to the promoter [12,47,48]. To date, only one example of a 5’/3’ gene loop has been thoroughly described in Arabidopsis at the *FLC* locus. At this specific locus, we showed that BAF60 interacts with the 5’ and the 3’ part of the *FLC* locus [12]. Indeed, BAF60 regulates *FLC* by negatively affecting the loop dynamics through the modulation of histone density, composition and post-translational modification. Similarly here we report modifications of the chromatin status of *IPT* and *KRP7* genes. Altogether, these data suggest that BAF60 is a general regulator of transcription through its ability to modulate gene loop structure, histone marks and RNA pol II deposition. One crucial question is the sequence of events that leads to these changes in chromatin organization: do modifications of histone marks allow gene loop formation and release, or does gene looping influence histone mark deposition? In yeast and mammals, certain repressive SWI/SNF-related complexes were shown to possess histone deacetylase activity, known to repress transcription, suggesting that nucleosome-remodelling activities could be coupled with chromatin modifying activities to promote transcriptional repression [49,50,51]. Our results support a model in which BAF60 containing complexes could possess both chromatin remodelling and histone modifying activities to promote transcriptional repression.

**Fig 6:**
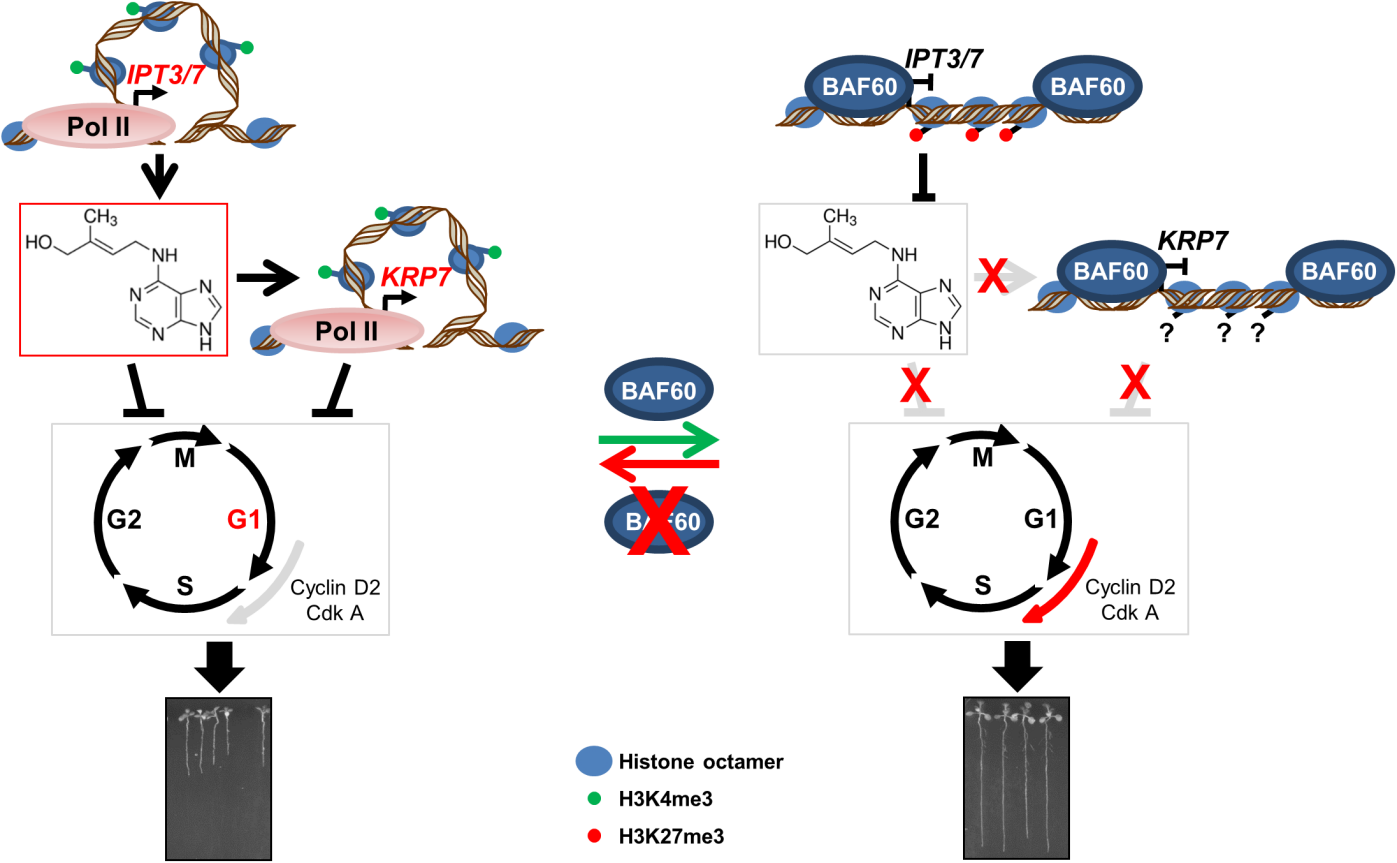
Model for the role of BAF60 in root development. In the wild-type root tip, BAF60 binds *IPT3*, *IPT7* and *KRP7* and inhibits their expression during root development. In contrast, in *BAF60* RNAi lines, these genes are overexpressed due to formation of gene loops and augmentation of H3K4me3 level. This blocks the cell cycle in G1 phase and inhibits root development.

In conclusion, SWI/SNF complexes containing BAF60 act on the expression of genes controlling CK production and cell cycle progression through the induction of histone modifications and control of gene looping structure (Fig 6). Changes in chromatin and epigenetic status of hormone biosynthetic genes and/or cell cycle regulators add a novel regulatory mechanism acting in plant growth.

After 15-min fixation in 100% cold acetone, β-glucuronidase (GUS) activity was revealed as described previously (Ni et al., 2009).

## Supporting Information

S1 Fig Quantification of root meristem lenght of five-day-old wild type and *BAF60* RNAi lines growth vertically on MS medium. Values are average +/- standard deviation (n > 15).
(TIF)

S2 Fig Real-time quantitative RT-PCR data showing relative expression of *Cyclin B1;1* in wild-type and *BAF60* RNAi roots. Total RNA samples were collected from 14-day-old roots. Data presented here are average +/- standard deviation obtained from three independent biological replicates. Asterisks indicate significant difference from the wild type (WT) (P < 0.01, Student’s t test).
(TIF)

S3 Fig Zeatin content is reduced in BAF60 RNAi lines. Zeatin 7 glucoside and zeatin 9 glucoside content in the root tips of 14-day-old wild-type and *BAF60* RNAi plantlets grown vertically on MS medium. Values are average +/- standard deviation of four biological replicates. Asterisks indicate significant difference from the wild type (WT) (P < 0.01, Student’s t test).
(TIF)

S4 Fig qRT-PCR data show the relative expression of the indicated genes in wild-type and *BAF60* RNAi lines. Total RNA samples were collected from 14-day-old roots. mRNA abundance was quantified by qRT-PCR and expressed relative to the abundance of UBQ10 transcripts. Values are average +/- standard deviation and were obtained on triplicates. Data presented are representative of three biological replicates.
(TIF)

S5 Fig *KRP7* gene expression is altered in BAF60 RNAi lines. Real-time quantitative RT-PCR data showing relative expression of *KRP7* in wild-type and *BAF60* RNAi roots. Total RNA samples were collected every 4 hr from 14-day-old roots. Grey areas behind the traces represent night periods. Data presented here are average +/- standard deviation obtained from three biological replicates. Asterisks indicate significant difference from the wild type (WT) (P < 0.01, Student’s t test).
(TIF)

S6 Fig BAF60 binds to the *KRP7* locus. (A) Schematic representation of the *KRP7* locus, the position of each primer pair used for ChIP-qPCR is indicated. The arrow indicates the position of the transcription start site. Exons are represented as black boxes and introns as black lines. (B) Quantification data of chromatin immunoprecipitation results. Nuclei were extracted after cross-linking from 14-day-old roots, expressing the BAF60-CFP fusion protein, sonicated, and chromatin-protein complexes were immuno-precipitated with antibodies directed against GFP or IgG. Average relative quantities ± sd are shown for each sample. Data presented are representative of three biological replicates. Asterisks indicate significant difference from the wild type (WT) (P < 0.05, Student’s t test).
(TIF)

S7 Fig BAF60 controls histone modifications and RNA Pol II occupancy at the *KRP7* loci. (A) Schematic representation of the regions of the *KRP7* locus analysed. Black boxes correspond to exons, the arrow indicates the site of translation initiation, numbers indicate the position of primer pairs used. (B) Quantification data of the chromatin immunoprecipitation results. Nuclei were extracted from 14-day-old roots and immunoprecipitation was performed with antibodies specific for H3K4me3 or RNA polymerase II. Average relative quantities ± sd are shown for each sample. Data presented are representative of three biological replicates. Asterisks indicate significant difference from the wild type (WT) (P < 0.05, Student’s t test).
(TIF)

S8 Fig BAF60 regulates gene loop formation at *KRP7* locus. Quantitative 3C of the *KRP7* locus, using region I as the anchor region, in 14-day-old WT and *BAF60* RNAi roots. Relative interaction frequencies were calculated as described in Materials and Methods. Data are the average of three biological replicates each with three technical replicates. In the graph, MfeI restriction sites are indicated with vertical dotted lines. A schematic representation of these loci is shown above with the position of primers used for the 3C analysis represented by grey arrowheads. Grey Asterisks indicate significant difference from the no crosslink condition (-) (P < 0.05, Student’s t test). Black Asterisks indicate significant difference from the wild type (WT) (P < 0.05, Student’s t test).
(TIF)

S1 Table Sequences of primers used in this study (5’-3’). (TIF)
