# Supplementary figures and images for: A SWI/SNF Chromatin Remodelling Protein Controls Cytokinin Production through the Regulation of Chromatin Architecture
Source: PLoS One. 2015 Oct 12;10(10):e0138276. doi: 10.1371/journal.pone.0138276 (PMC4601769; doi:10.1371/journal.pone.0138276)

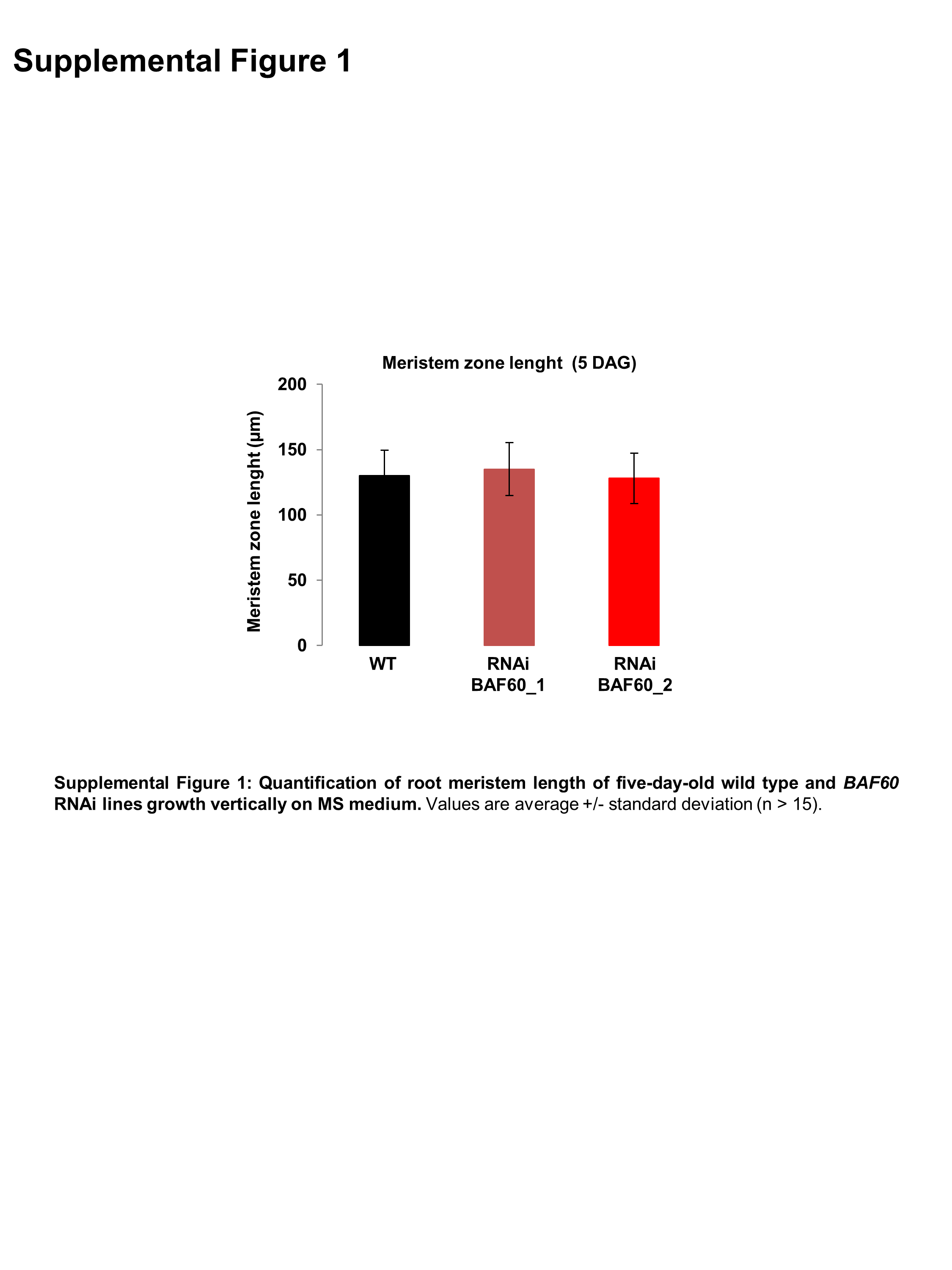

Supplement: S1 Fig — Values are average +/- standard deviation (n > 15). (TIF) [file pone.0138276.s001.TIF]

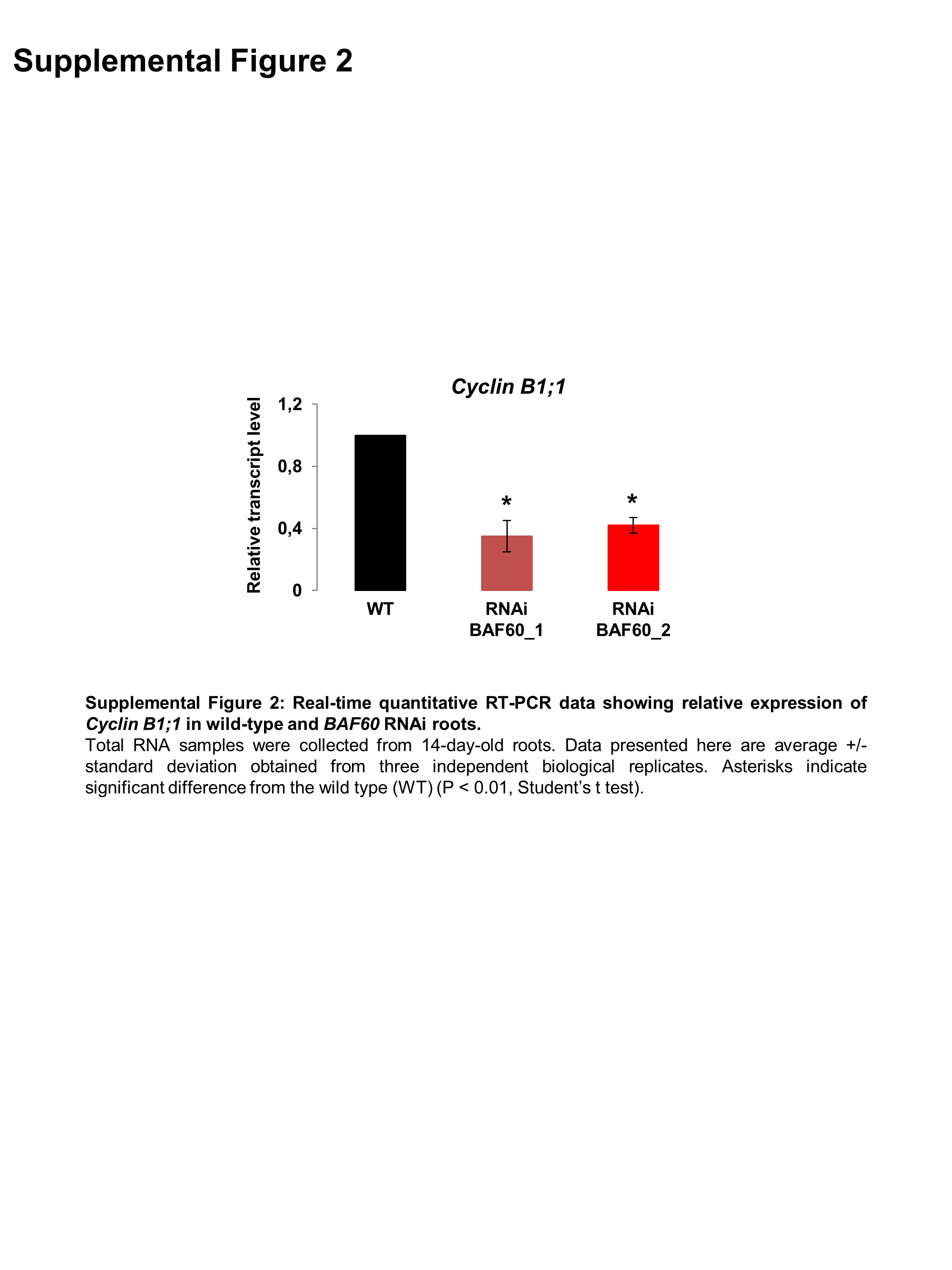

Supplement: S2 Fig — Total RNA samples were collected from 14-day-old roots. Data presented here are average +/- standard deviation obtained from three independent biological replicates. Asterisks indicate significant difference from the wild type (WT) (P < 0.01, Student’s t test). (TIF) [file pone.0138276.s002.TIF]

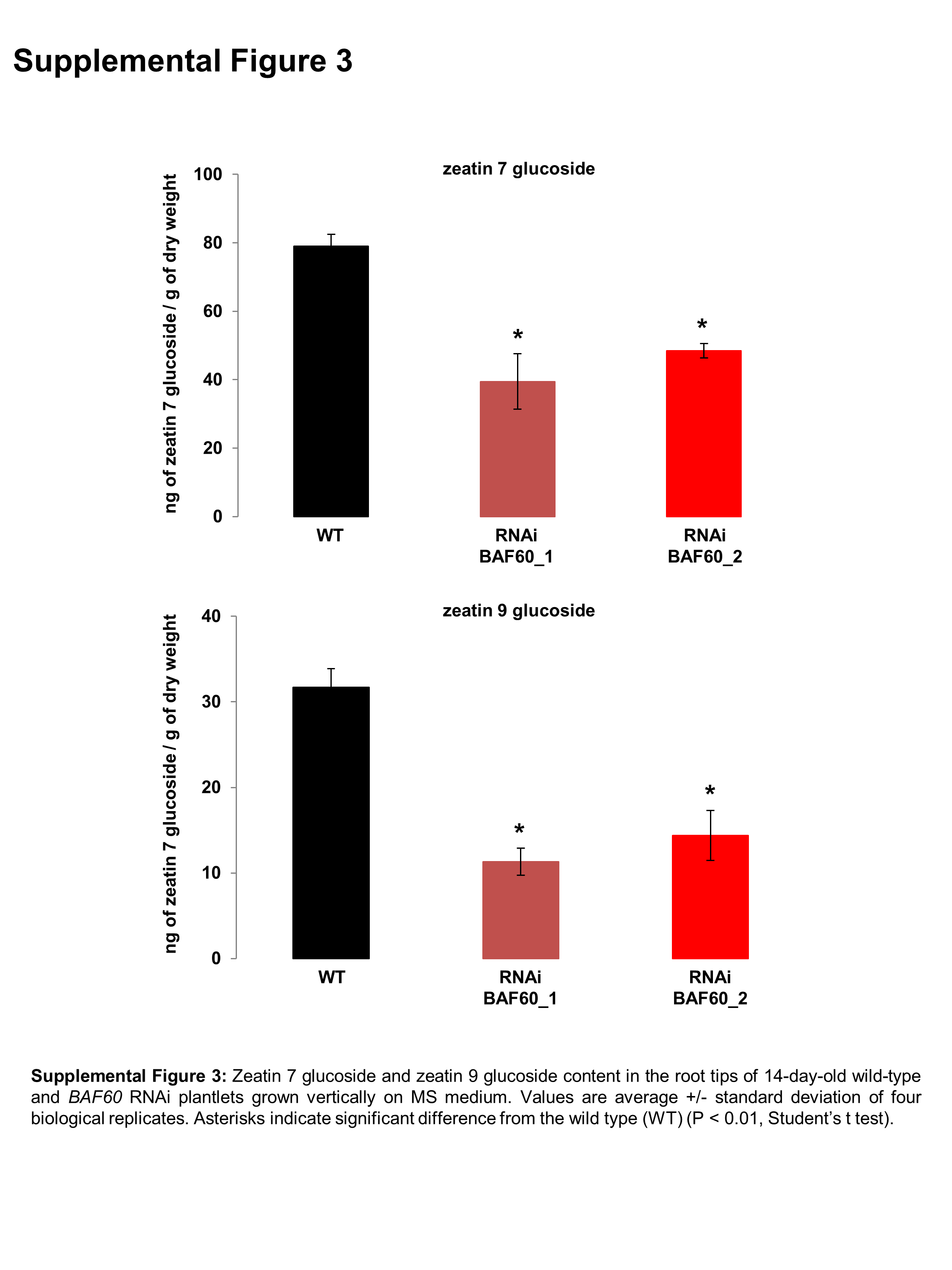

Supplement: S3 Fig — Zeatin 7 glucoside and zeatin 9 glucoside content in the root tips of 14-day-old wild-type and BAF60 RNAi plantlets grown vertically on MS medium. Values are average +/- standard deviation of four biological replicates. Asterisks indicate significant difference from the wild type (WT) (P < 0.01, Student’s t test). (TIF) [file pone.0138276.s003.TIF]

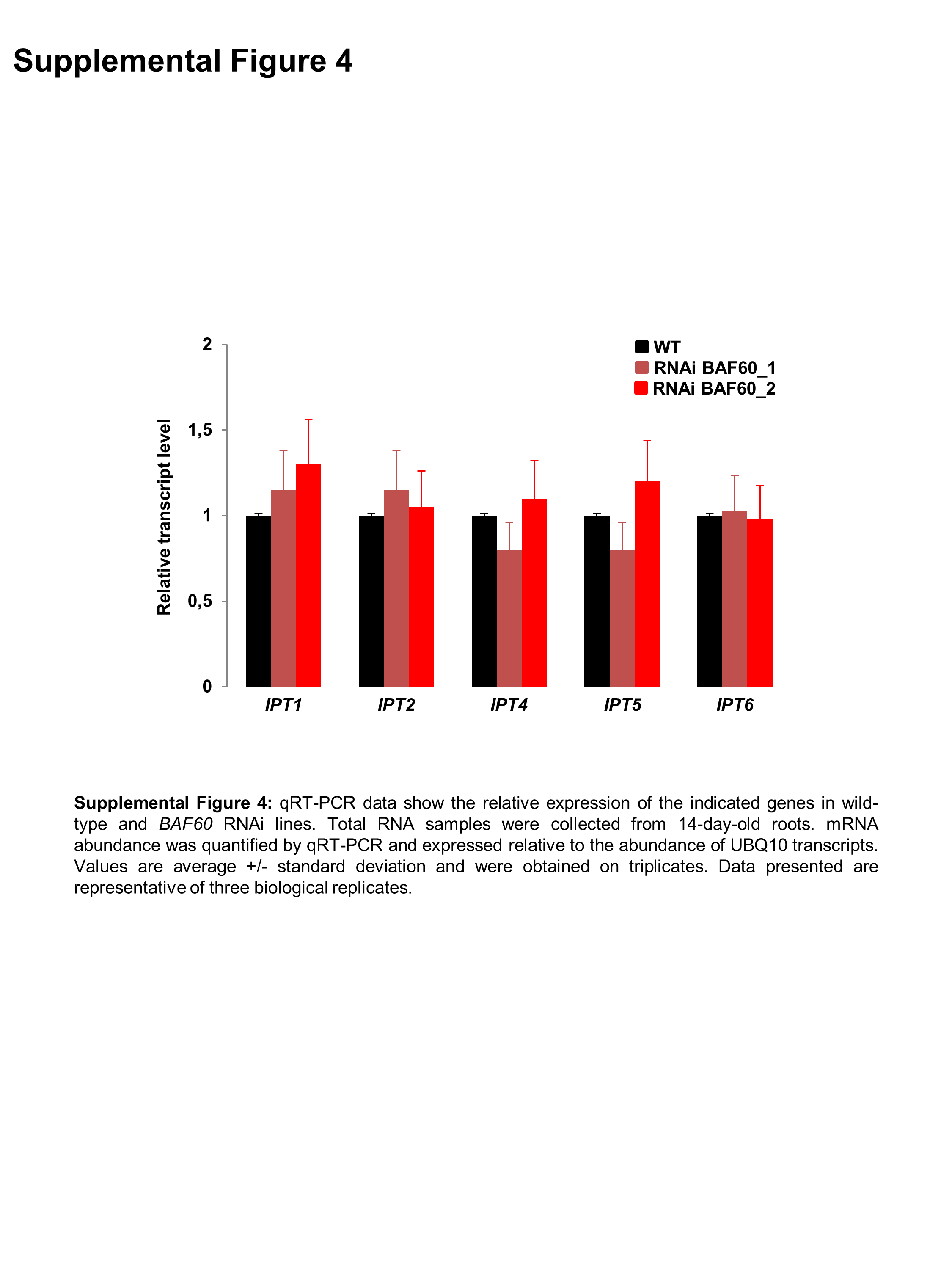

Supplement: S4 Fig — Total RNA samples were collected from 14-day-old roots. mRNA abundance was quantified by qRT-PCR and expressed relative to the abundance of UBQ10 transcripts. Values are average +/- standard deviation and were obtained on triplicates. Data presented are representative of three biological replicates. (TIF) [file pone.0138276.s004.TIF]

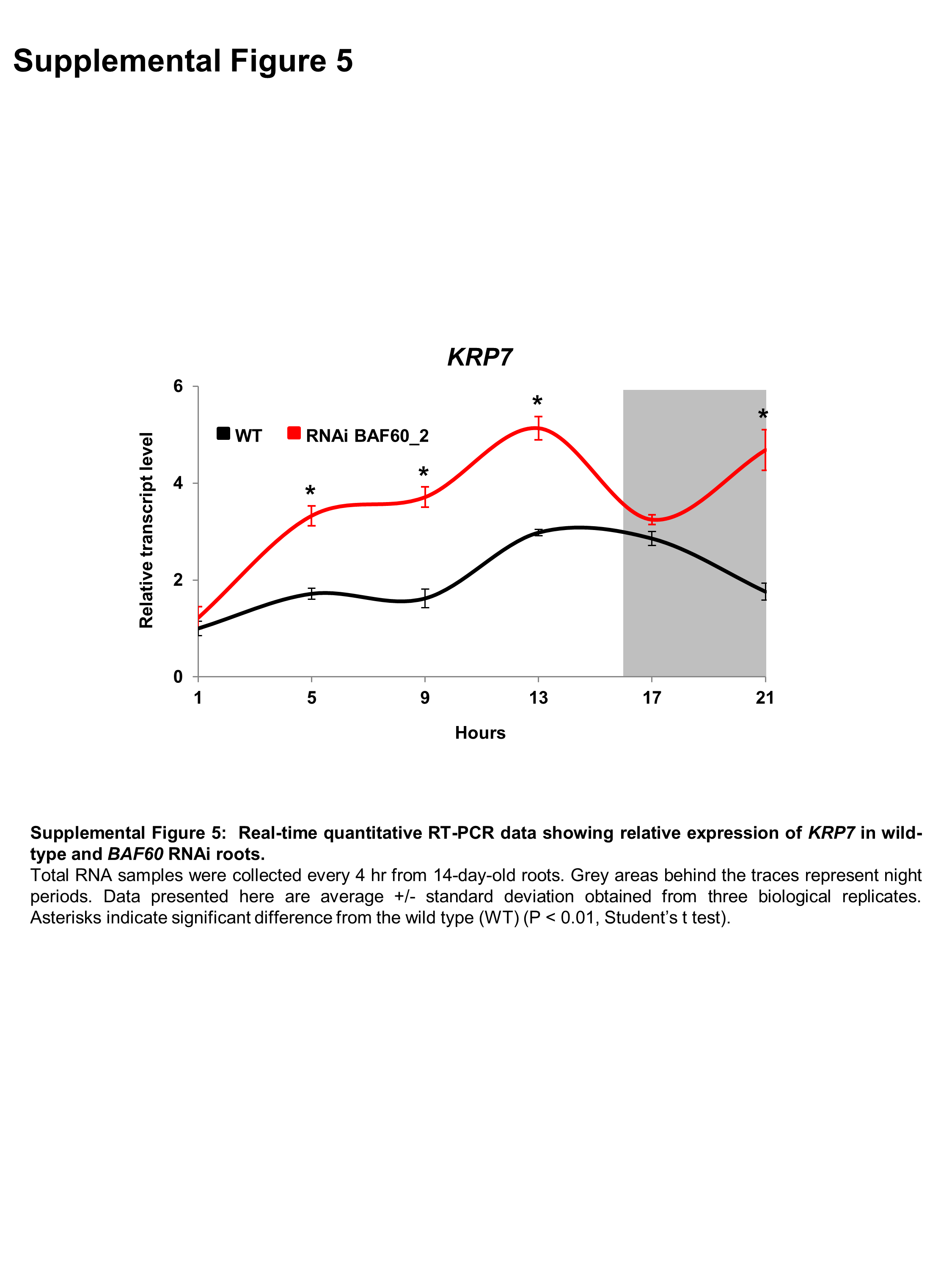

Supplement: S5 Fig — Total RNA samples were collected every 4 hr from 14-day-old roots. Grey areas behind the traces represent night periods. Data presented here are average +/- standard deviation obtained from three biological replicates. Asterisks indicate significant difference from the wild type (WT) (P < 0.01, Student’s t test). (TIF) [file pone.0138276.s005.TIF]

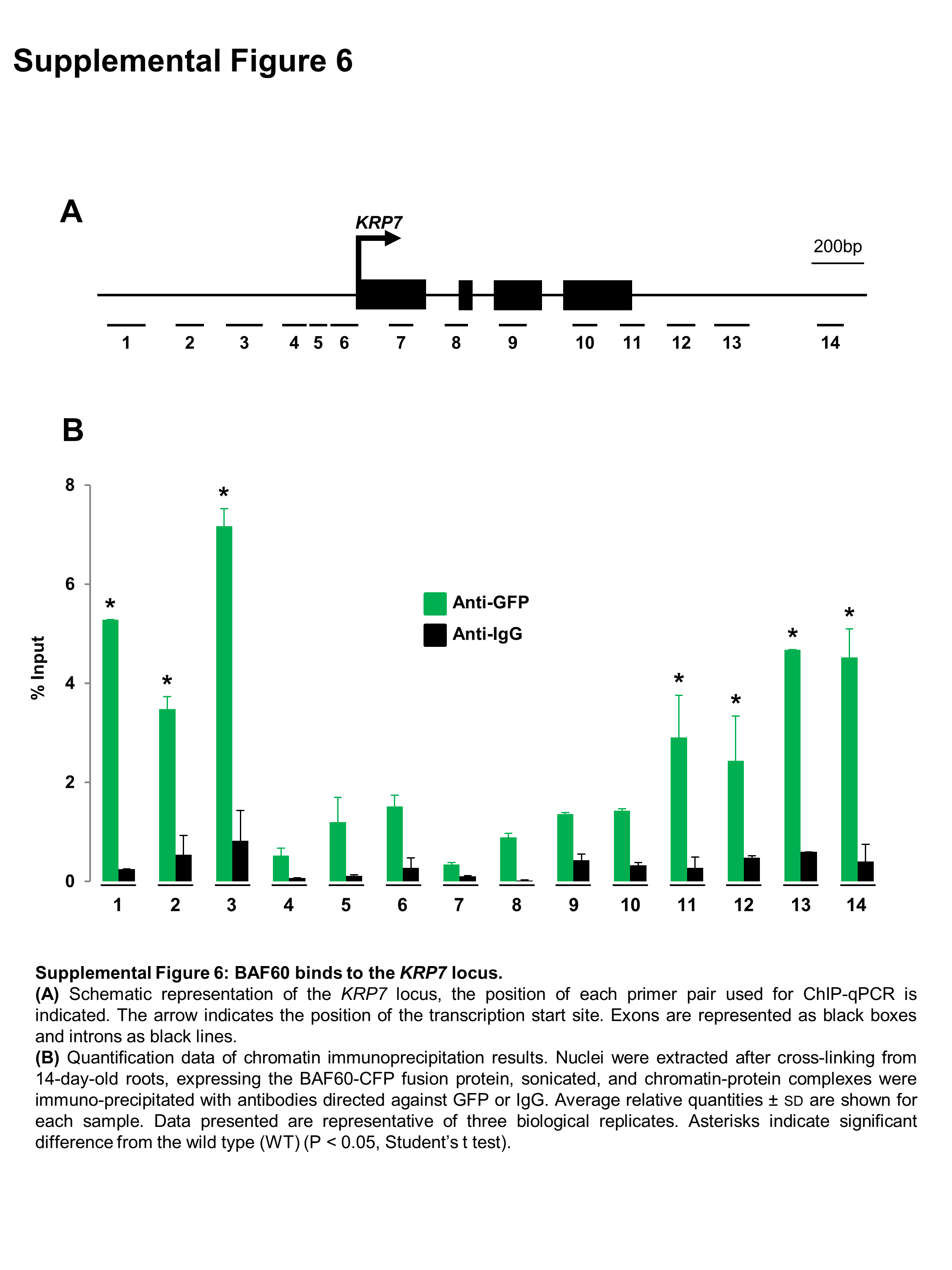

Supplement: S6 Fig — (A) Schematic representation of the KRP7 locus, the position of each primer pair used for ChIP-qPCR is indicated. The arrow indicates the position of the transcription start site. Exons are represented as black boxes and introns as black lines. (B) Quantification data of chromatin immunoprecipitation results. Nuclei were extracted after cross-linking from 14-day-old roots, expressing the BAF60-CFP fusion protein, sonicated, and chromatin-protein complexes were immuno-precipitated with antibodies directed against GFP or IgG. Average relative quantities ± sd are shown for each sample. Data presented are representative of three biological replicates. Asterisks indicate significant difference from the wild type (WT) (P < 0.05, Student’s t test). (TIF) [file pone.0138276.s006.TIF]

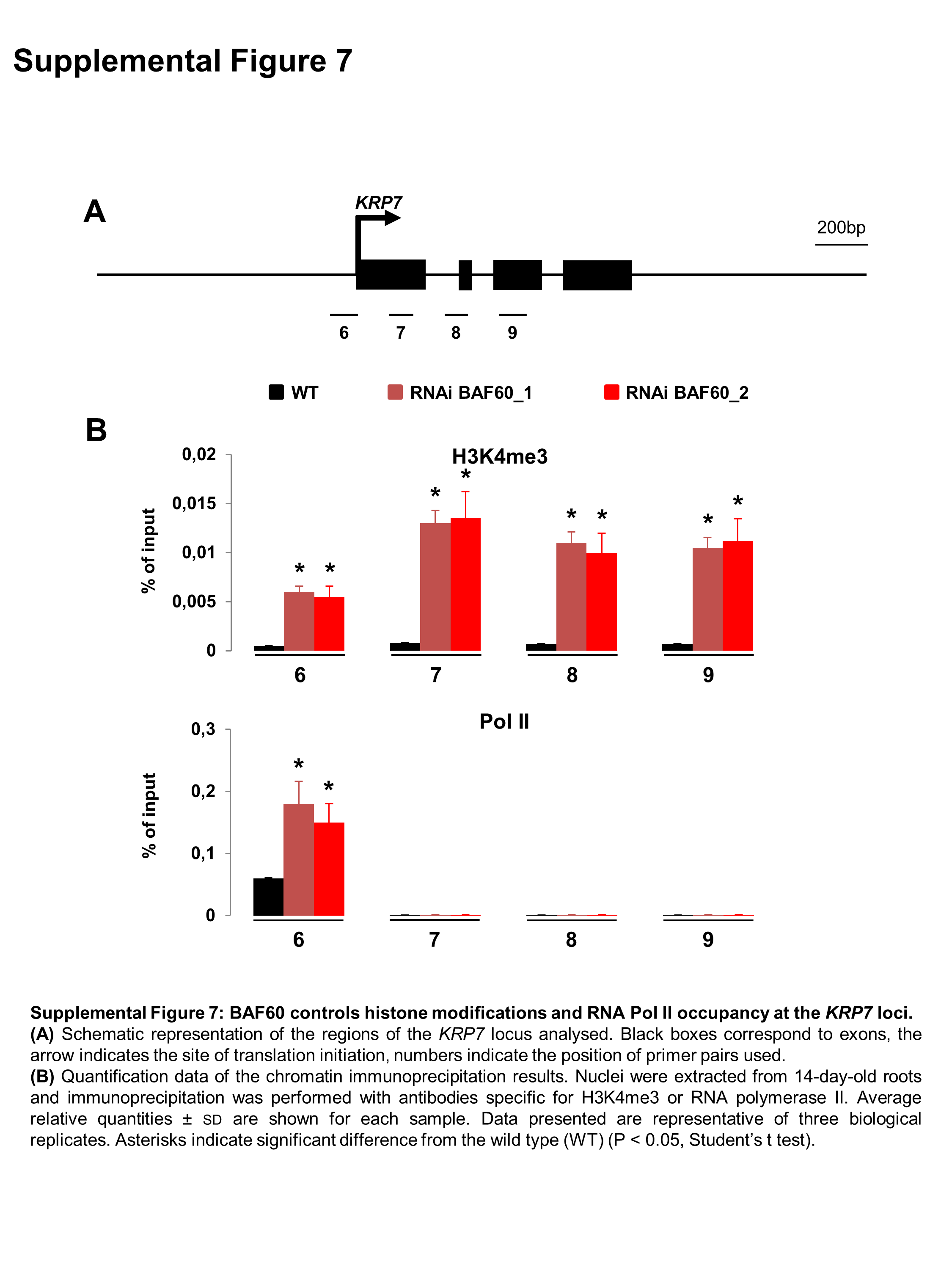

Supplement: S7 Fig — (A) Schematic representation of the regions of the KRP7 locus analysed. Black boxes correspond to exons, the arrow indicates the site of translation initiation, numbers indicate the position of primer pairs used. (B) Quantification data of the chromatin immunoprecipitation results. Nuclei were extracted from 14-day-old roots and immunoprecipitation was performed with antibodies specific for H3K4me3 or RNA polymerase II. Average relative quantities ± sd are shown for each sample. Data presented are representative of three biological replicates. Asterisks indicate significant difference from the wild type (WT) (P < 0.05, Student’s t test). (TIF) [file pone.0138276.s007.TIF]

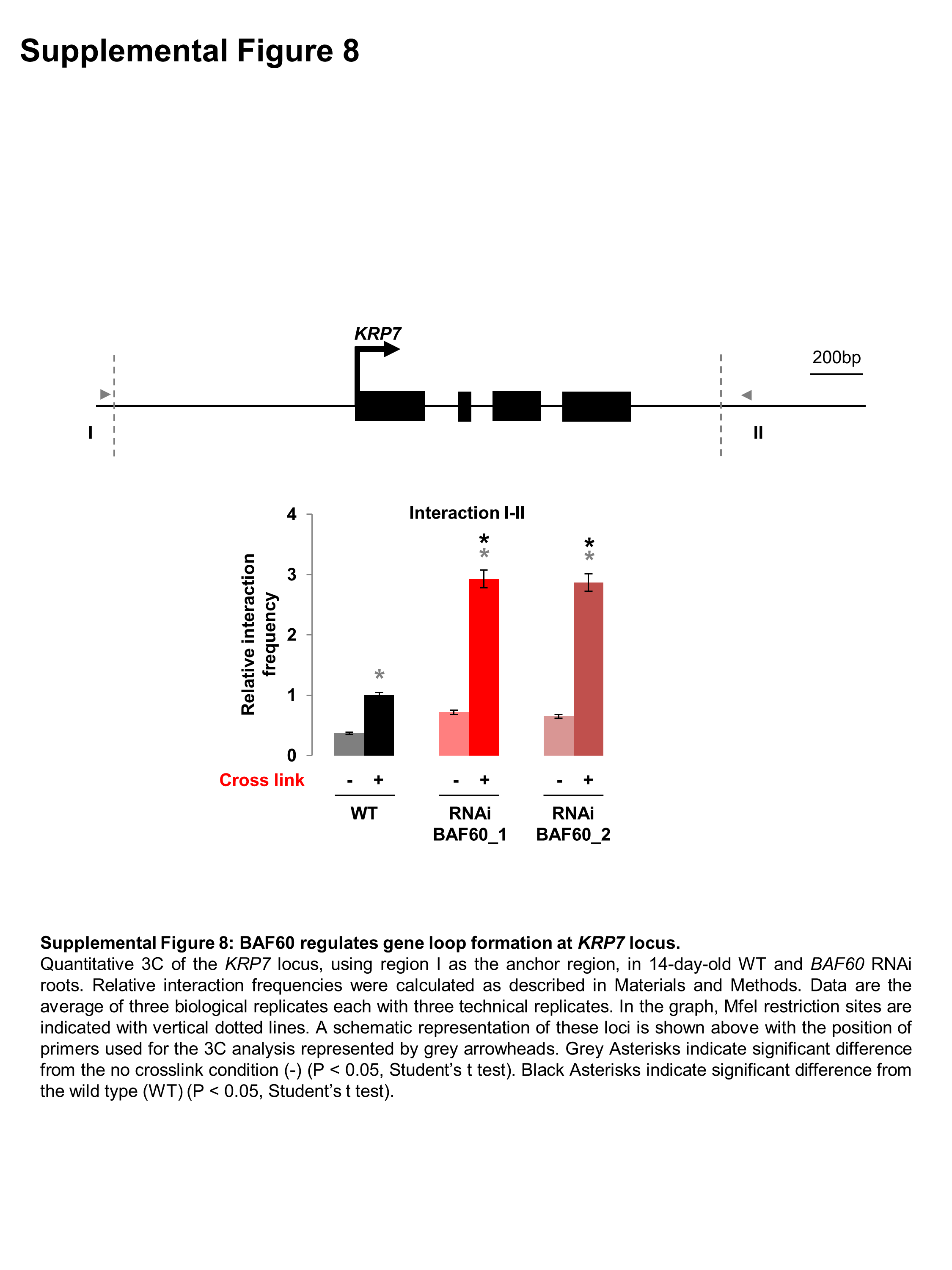

Supplement: S8 Fig — Quantitative 3C of the KRP7 locus, using region I as the anchor region, in 14-day-old WT and BAF60 RNAi roots. Relative interaction frequencies were calculated as described in Materials and Methods. Data are the average of three biological replicates each with three technical replicates. In the graph, MfeI restriction sites are indicated with vertical dotted lines. A schematic representation of these loci is shown above with the position of primers used for the 3C analysis represented by grey arrowheads. Grey Asterisks indicate significant difference from the no crosslink condition (-) (P < 0.05, Student’s t test). Black Asterisks indicate significant difference from the wild type (WT) (P < 0.05, Student’s t test). (TIF) [file pone.0138276.s008.TIF]

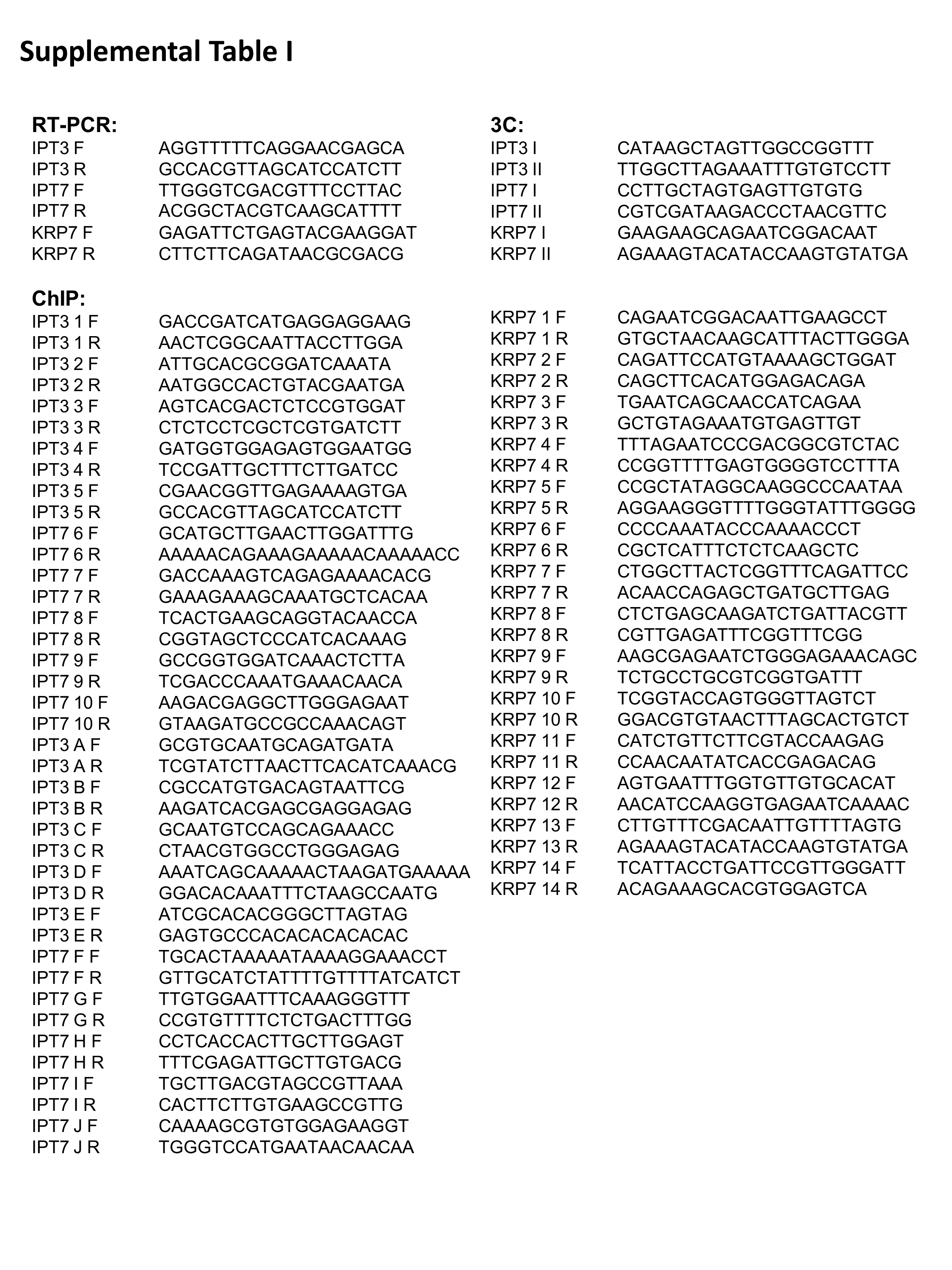

Supplement: S1 Table — (TIF) [file pone.0138276.s009.TIF]
